# Immunosuppressive Therapy in Patients with Aplastic Anemia: A Single-Center Retrospective Study

**DOI:** 10.1371/journal.pone.0126925

**Published:** 2015-05-13

**Authors:** Hasan Jalaeikhoo, Ahmad Khajeh-Mehrizi

**Affiliations:** AJA cancer research center (ACRC), AJA University of Medical Sciences, Tehran, Iran; University Hospital of Salamanca, SPAIN

## Abstract

**Background:**

Aplastic anemia (AA) is a rare disease in which hematopoietic stem cells are severely diminished resulting in hypocellular bone marrow and pancytopenia. Etiology of AA includes auto immunity, toxins, infection, ionizing radiation, drugs and rare genetic disorders, but in the majority of cases no cause can be identified. In the present study we assessed response rate, survival, relapse and clonal evolution in patients with AA treated with immunosuppressive therapy.

**Methods:**

Patients with AA who received immunosuppressive therapy between May 1998 and September 2013 were included in this study. Patients with non-severe AA (NSAA) were treated with cyclosporine (CsA) and danazol while patients with severe AA (SAA) as well as patients with NSAA who progressed to SAA after beginning of the treatment, were candidates for receiving antithymocyte globulin in addition to CsA and danazol.

**Results:**

Among the 63 studied patients, 29 (46%) had NSAA and 34 (54%) had SAA. Three months after treatment, overall response was 58.6% in NSAA and 12.9% in patients with SAA. Survival of all patients at 5, 10 and 15 years were 73%, 55% and 49%, respectively. Survival rates were significantly higher in patients with NSAA compared to patients with SAA as well as in patients who responded at 6 months compared to non-responders. The relapse risk was 39.7% at 10 years. Relapse occurred in patients who discontinued the therapy more than those who continued taking CsA (p value<0.01). The risk of clonal evolution was 9.9% at 10 years and 22.8% at 15 years after treatment.

**Conclusion:**

This long-term retrospective study indicated that immunosuppressive therapy should be recommended to patients with AA. Also, our experience indicated that immunosuppressive therapy should not be discontinued after response to therapy in patients with both NSAA and SAA due to high risk of relapse. Low dose of CsA should be continued indefinitely.

## Introduction

Aplastic anemia (AA) is a rare disease which is characterized by peripheral pancytopenia and hypocellular bone marrow in the absence of malignant infiltration or hyperplasia of reticulin fibers. The incidence of AA in American and European countries is around 0.23 per 100000 population per year [1] while its incidence increases to 0.39–0.50 per 100000 population per year in Asian countries [2, 3]. AA can be either inherited or acquired. Toxins, infections, ionizing radiation, drugs, rare genetic causes with congenital deformities and nutritional deficiencies have been identified as etiologic factors. Thymoma and auto immunities are identified as the etiology in other cases [4, 5]. The vast majorities of cases with acquired AA have no definite causative agents and are classified as idiopathic.

AA may progress to acute leukemia or myelodisplastic syndrome. Paroxysmal nocturnal hemoglobinuria may be a concomitant disease with AA. However, for the most part, AA behaves like an immune-mediated disease. Clinical manifestations of AA depending on the peripheral blood cell count may include symptoms such as fatigue, easy bruising, petechiae, epistaxis, gingival bleeding, headache and fever. A minority of patients are suitable candidates for HSCT. Advanced age, lack of histocompatible sibling donor and high costs makes HSCT unavailable for patients. Immunosuppressive therapy (IST) is an effective alternative in such patients that results in a satisfactory response [6–10].

There is no data on the outcomes of the IST in patients with AA in Iran. In Iran, the treatment options are more limited due to socioeconomically disadvantaged status of many patients which could impact overall survival. The present single-center study set in Iran is aimed at retrospectively analyzing the response rate, survival, relapse and clonal evolution in patients treated with IST.

## Materials and Methods

### Cohort

All patients with AA who received IST at Imam Reza hospital in Tehran between May 1998 and September 2013 were identified. Patients were included in this retrospective study if they fulfilled the criteria for AA and were not transplanted due to advanced age or lack of availability of a histocompatible sibling donor. Suitable candidates for transplantation who refused HSCT were also included in this study. Exclusion criteria included congenital AA, bone marrow findings consistent with myelodysplastic syndrome (MDS) or early recovery from pancytopenia (within 90 days after diagnosis). Baseline laboratory values were defined as the lowest value prior to IST to exclude transfusion artifacts and included hemoglobin level, platelet count, absolute neutrophil count (ANC), absolute lymphocyte count (ALC) and absolute reticulocyte count (ARC). Bone marrow flow cytometry to rule out the other causes of pancytopenia and serology examination for hepatitis A, B, C, E, Epstein barr virus (EBV) and Parvovirus B19 was carried out in every patients. This study was approved by the ethics committee of the AJA Cancer Research Center (ACRC) and the institutional review board of the AJA University of Medical Sciences. Written informed consent was obtained from each patient to allow the use of their medical records for research. Data were obtained from medical records.

### Definitions

Severe AA (SAA) was defined by the presence of a hypocellular bone marrow (cellularity less than 30%) and 2 of the 3 following criteria: platelet counts<20*10^9^/L, absolute neutrophil count (ANC)<0.5*10^9^/L, and absolute reticulocyte count (ARC) <20*10^9^/L [11]. Patients with ANC<0.2*10^9^/L were classified as very SAA (VSAA) [12]. In this study, three patients had VSAA that were classified as SAA in the analysis. Patients with AA who did not meet criteria for SAA but with at least 2 of the 3 following parameters (ANC < 1*10^9^/L, platelet counts < 50*10^9^/L, and ARC < 60*10^9^/L), were considered as non-severe AA (NSAA). Complete response (CR) was defined as achieving an ANC > 1.5 * 10^9^/L, platelet count > 100 * 10^9^/L, and hemoglobin (Hb) > 100 g/L. Partial response (PR) was defined by transfusion independence and an increase in hematological parameters from baseline values with at least Hb > 70 g/l, ANC > 0.5×10^9^/l and platelet count > 30×10^9^/l. A patient was considered in relapse if blood counts decreased; and consequently he or she required transfusion of red blood cells or platelets and reinstitution of immunosuppressive therapy. Clonal evolution was defined when a new clonal disorder appeared on cytogenetic analysis or bone marrow analysis showed the characteristic morphologic changes.

### Treatment protocol

Patients with NSAA were treated with cyclosporine (CsA) and danazol. Patients with SAA or VSAA as well as patients with NSAA who progressed to SAA after beginning of the treatment were candidates for receiving antithymocyte globulin (ATG) in addition to CsA and danazol. Some patients did not receive ATG due to episodic unavailability of this medicine in Iran; hence such patients were given only CsA and danazol or were given ATG several months after beginning the treatment of AA.

CsA was given 6 mg/kg per day orally, continued for at least 6 months adjusted to blood levels (therapeutic range from 150 to 250 ng/ml). In responders with a stable blood counts (absolute neutrophil count, hemoglobin level and platelet count), CsA was gradually tapered (1mg/kg every 2 weeks) if hematologic parameters remained stable during the course of tapering. We prescribed CsA at a dose of 1–2 mg/kg indefinitely for maintenance therapy. Danazol was administered at 5–10 mg/kg per day orally.

An ATG skin test was performed on all patients to assess immediate hypersensitivity. Horse ATG (Atgam, Pharmacia & Upjohn Company, Kalamazoo, Mich) at a dose of 15 mg/kg per day was intravenously administered on day1–8. Dexamethasone 16 mg per day (divided q12hr) was given intravenously on day 1–8 for prevention of serum sickness.

### Statistical analysis

To compare the difference between continuous variables Mann–Whitney U test and between categorical variables the χ2-test were used. Logistic regression was used to assess the multiple factors that affect the response to therapy. Survival analyses were done by the Kaplan–Meier method and compared by the log-rank test. Cumulative incidence was analyzed using the Kaplan-Meier method for time to relapse among patients who achieved to response and time to evolution for all patients. The Cox proportional hazards model was used to examine the potential prognostic factors that affect survival, relapse and clonal evolution. P-value less than 0.05 were considered to be statistically significant. PASW version 18 was used to perform all statistical analyses.

## Results

### Patients’ characteristics

Among the 63 studied patients, 29 (46%) had NSAA and 34 (54%) had SAA. Twelve (19%) patients treated with ATG, CsA and danazol while 51 (81%) patients received CsA and danazol. Three patients had hepatitis associated AA; two were positive for hepatitis C and one had seronegative hepatitis. Two patients were positive for parvovirus B19. One patient was a house painter, two patients worked at chemical industries and one patient had history of taking penicillamine. Characteristics of studied patients are shown in Table 1.

**Table 1:** Characteristics of studied patients.

characteristic	NSAA (N = 29)	SAA (N = 34)
Age (year)	32.2 (16.6)	30 (13.4)
Sex		
Male	19 (65.5)	22 (64.7)
Female	10 (34.5)	12 (35.3)
Etiology		
Idiopathic	24 (82.7)	30 (88.2)
Hepatitis associated	1 (3.4)	2 (5.8)
Parvovirus B19	2 (6.8)	0
Chemical & Drug induced	2 (6.8)	2 (5.8)
Baseline		
Hemoglobin (g/L)	90.8 (17)	71.9 (14.1)
Platelet count (×10^9^/L)	27.7(13.3)	12.4 (5.2)
Absolute neutrophil count (×10^9^/L)	0.68 (0.19)	0.41 (0.19)
Absolute lymphocyte count (×10^9^/L)	1.81 (0.51)	1.33 (0.54)
Absolute reticulocyte count (×10^9^/L)	26.8 (7.1)	14.5 (5.1)

Data are presented as mean (SD) except sex and etiology which are presented as n (%).

NSAA non-severe aplastic anemia, SAA severe aplastic anemia

### Response to therapy

The CR and overall response (CR+PR) of the patients with NSAA were significantly higher (p value <0.001) than patients with SAA at 3, 6 and 12 months after treatment (Table 2). We did not compare the response between patients received the ATG and who did not, because for majority of patients, ATG was administered several months after beginning the treatment. ANC and platelet count were significantly associated with overall response at 3 months after treatments (p value = 0.03 and 0.005, respectively). No factor was significantly associated with overall response at 6 and 12 months after treatment.

**Table 2:** Response to treatment and death at 3, 6 and 12 months after treatment.

Time after treatment	NSAA (N = 29)	SAA (N = 34)	P value
3 months			
No. of patients	29	31	
Complete Response	3 (10.3)	0	<0.001
Partial Response	14 (48.3)	4 (12.9)	
Overall Response	17 (58.6)	4 (12.9)	<0.001
Death	0	4 (12.9)	
6 months			
No. of patients	28	29	
Complete Response	11 (37.9)	2 (6.8)	<0.001
Partial Response	14 (48.3)	10 (34.4)	
Overall Response	25 (86.2)	12 (41.2)	<0.001
Death	0	6 (20.6)	
12 months			
No. of patients	27	25	
Complete Response	17 (62.9)	5 (20)	<0.001
Partial Response	8 (29.6)	8 (32)	
Overall Response	25 (91.5)	13 (52)	<0.001
Death	1 (3.7)	6 (24)	

Data are presented as n (%)

NSAA non-severe aplastic anemia, SAA severe aplastic anemia

Four patients died within 3 months, all had SAA. Three patients died from multiple organ failure due to sepsis and one patient died from pneumonia.

### Survival

**Fig 1:**
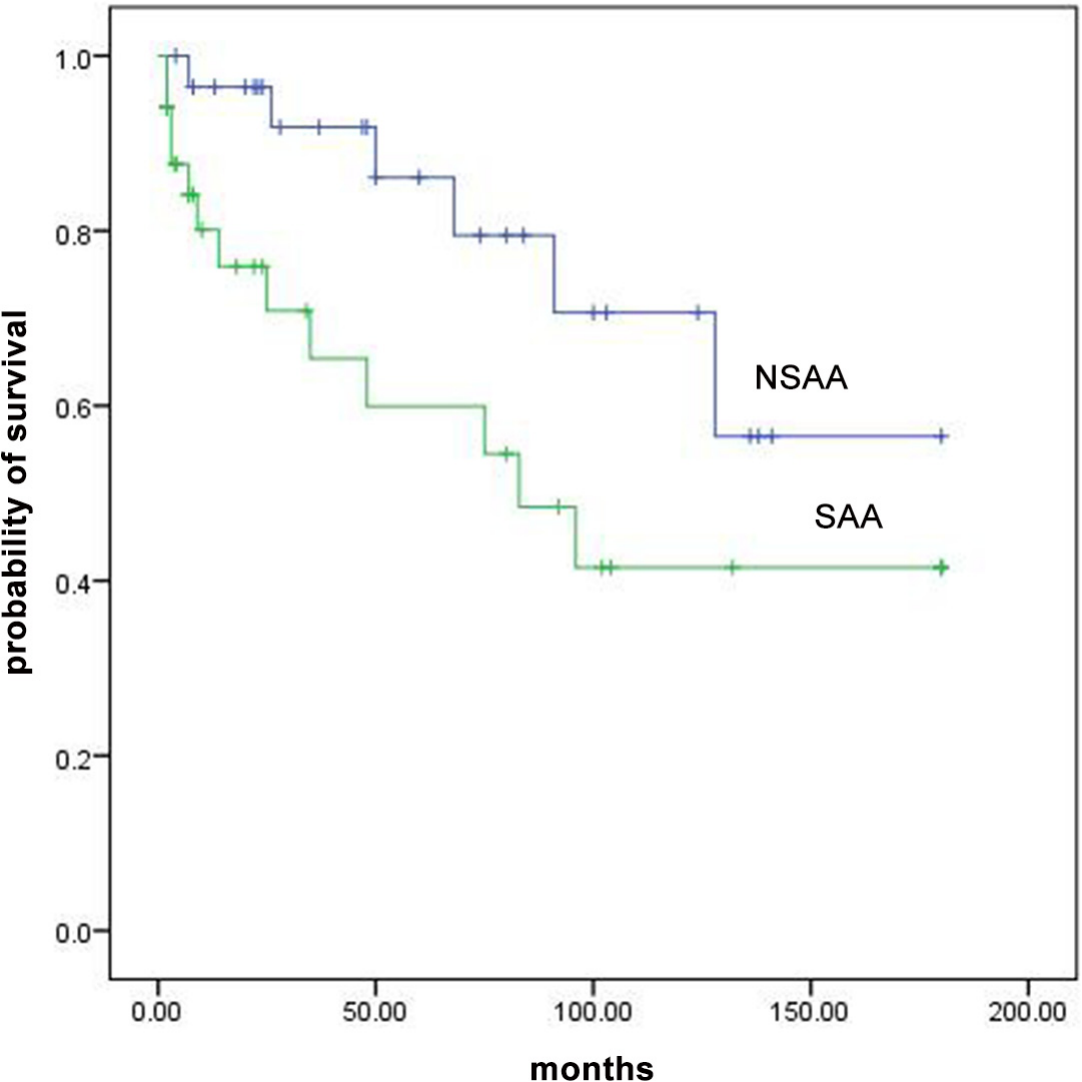
Overall survival curves for patients with SAA and NSAA

**Fig 2:**
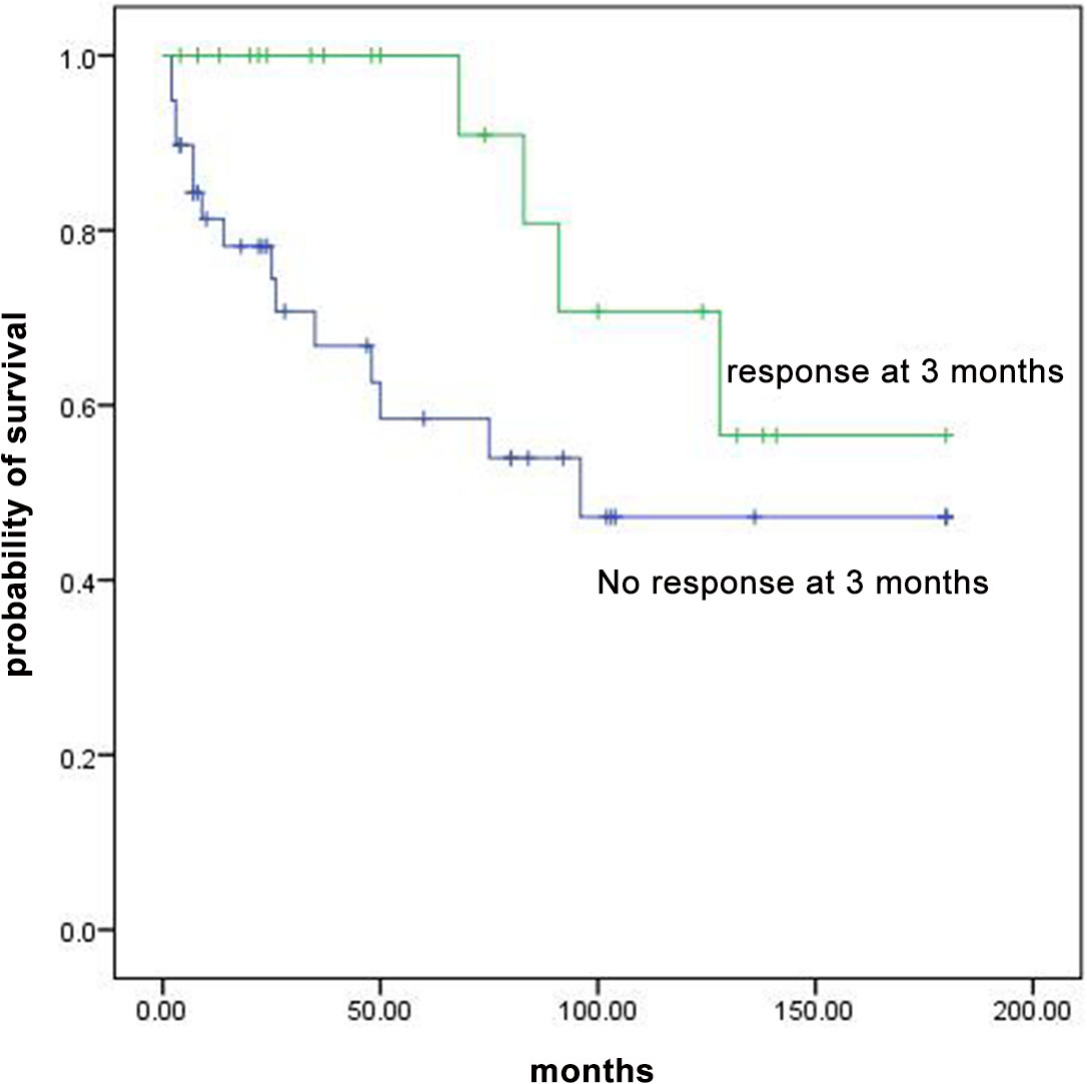
Overall survival curves for patients with AA who responded at 3 months and non responders.

**Fig 3:**
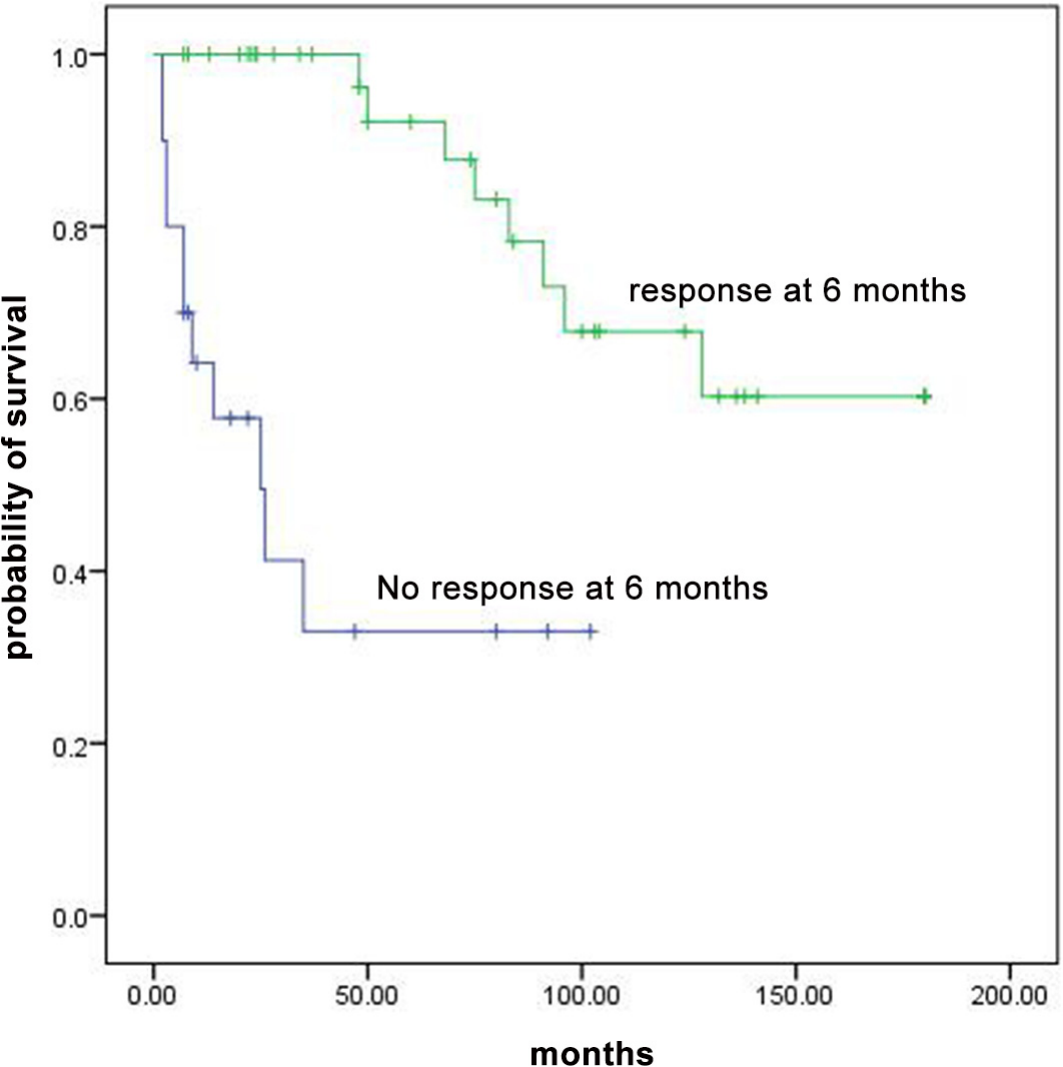
Overall survival curves for patients with AA who responded at 6 months and non responders.

In total, 19 patients died; 6 had NSAA and 13 had SAA; 14 treated with CsA and danazol and 5 treated with CsA, danazol and ATG. ATG side effect (long-standing neutropenia leading to sepsis) was considered as cause of death in one patient. In all patients, survival at 3, 5, 10 and 15 years were 78%, 73%, 55% and 49%, respectively. Survival was similar between patients who were treated with or without ATG (p value = 0.29). Survival rates at 3, 5, 10 and 15 years were significantly higher in patients with NSAA compare to patients with SAA (92% vs 65%, 86% vs 59%, 70% vs 41%, 56% vs 41%, p value = 0.04, Fig 1). Overall survival of patients who responded at 3 months was higher than non-responders but the difference was not statistically significant (P value = 0.06, Fig 2). For patients with overall response at 6 months, overall survival was statistically higher than non-responders (p value<0.001, Fig 3). Multivariable analysis showed no specific factor was significantly associated with the survival of patients.

### Relapse

**Fig 4:**
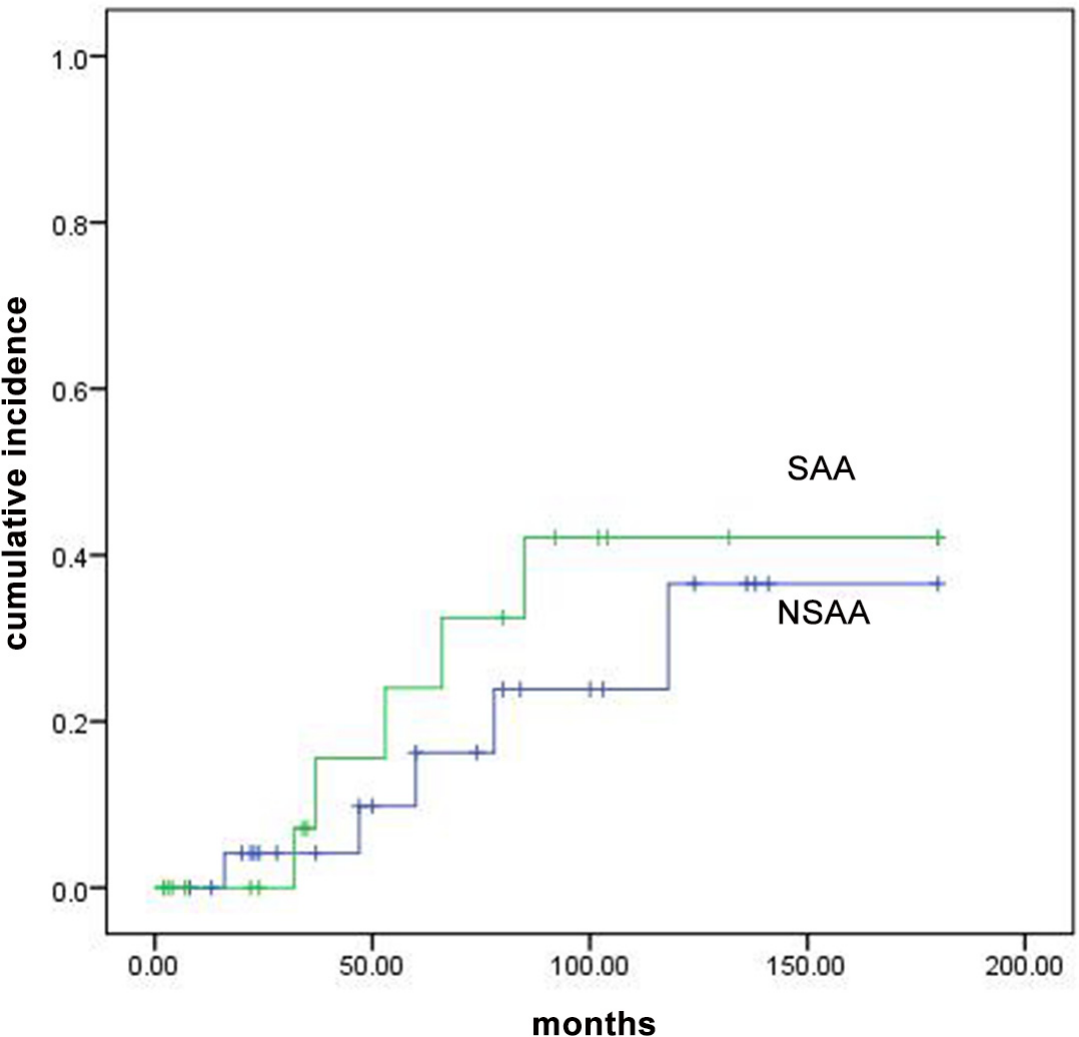
Cumulative incidence of relapse for patients with SAA and NSAA.

Among the patients who responded to treatment, 10 patients relapsed: five had NSAA and five had SAA. Two of them treated with ATG and eight were not. Relapses commonly occurred after discontinuation of therapy. Among 43 patients who responded to treatment, 12 discontinued the therapy which 7 of them relapsed; 31 continued the therapy which 3 of them relapsed (p value<0.01). The risk of relapse in all patients at 3, 5 and 10 years were 8.8%, 19.5% and 39.7% respectively. There was not a significant difference in relapsed cases between NSAA and SAA (36.6% vs. 43.1%, p value = 0.53, Fig 4). Multivariable analysis showed no specific factor was significantly associated with the relapse risk. Characteristics of 43 patients who were at risk of relapse are shown in S1 Table.

### Clonal evolution

Four patients showed the evidence of clonal evolution during study; three had NSAA and one had SAA. All patients previously achieved responses to treatments (3 in CR and 1 in PR) and all were treated without ATG. MDS developed in all four patients. Two patients developed acute myeloid leukemia evolving from MDS and died rapidly after supportive care. The risk of clonal evolution observed in the study was 9.9% at 10 years and 22.8% at 15 years after treatment.

## Discussion

Aplastic anemia can be treated with IST. Overall long term survival is comparable with either modality of HSCT or IST [13]. Several studies have demonstrated the successful outcome of the IST in patients who were not eligible for HSCT [13]. ATG based immune suppression has been advocated for improved remission induction and CsA as the standard maintenance of AA regimen for IST. In Iran, cost consideration of the drugs or procedures impacts treatment decisions. The numbers of patients who are candidates for HSCT or ATG are more than the numbers who receive these treatments. Therefore many patients with AA in our hospital only receive CsA and danazol. A study by the European Group for Blood and Bone Marrow Transplantation indicated that in 1999 approximately 50% of the patients who were eligible for IST were actually treated with ATG + CSA, while in 2002, 75% of patients treated with this therapy [14].

Our results are comparable with other studies reported in the literature. Comparisons of our findings with reports in the literature indicate that many patients with AA in Iran who did not receive ATG therapeutic regimens did relatively well. Gluckman et al. in a randomized controlled study found better result with CsA comparing to ATG but skill in treatment delivery and infection control gave superiority to ATG in later years [15]. Our results showed no influence from ATG infusion in our patients.

In this study, we assessed the outcomes of IST in AA patients over a long period of time. We reported the 15 years survival of AA patients were treated with IST and found that patients who responded at 6 months had significantly higher survival than non-responders. In our study, we found that relapse occurred in responders who discontinued CsA and found a moderately high risk of clonal evolution among early responders.

In our study, the response rates in NSAA patients were significantly higher than SAA. At 3, 6 and 12 months after treatment, overall response was 58.6%, 86.2% and 91.5% in NSAA patients respectively while overall response was 12.9%, 41.2% and 52% in SAA patients respectively. Our results are relatively similar to the study of Wang et al. [16] in China. They reported the overall response of CsA + androgen therapy and CsA + ATG therapy in SAA patients, 58.8% and 77.8% respectively while overall response of CsA + androgen therapy in NSAA patients was 81.5%. In their study one patient with NSAA received CsA +ATG therapy and CR was achieved. The overall response of ATG + CsA therapy for SAA varied between 55 and 87 percent in large studies [17].

We reported the long-term survival of patients with AA. Survival of all patients treated with IST in 10 and 15 years after treatment was 55% and 49% respectively. Frickhofen et al. [18] reported a survival of 58% at 11.3 years for patients treated with ATG with or without CsA. Rosenfeld et al. [19] reported the survival of 55% at 7 years for patients received ATG + CsA. In our study patients with overall response at 3 and 6 months had higher survival rates than non-responders but difference was only significant at 6 months. Among responders at 3 months, four patients died. Two deaths were associated with the development of acute myloid leukemia; this reduced the overall survival of responders at 3 months and made the difference not significant. In Rosenfeld et al. [19] study the difference of overall survival between responders at 3 months and non-responders was statistically significant.

We report the risk of 19.5% and 39.7% for relapse at 5 and 10 years respectively. similarly, Frickhofen et al. [18] reported the risk of 38% for relapse during 11.3 years and Sheinberg et al. reported cumulative incidence was 37% for relapse at 4 years in SAA patients received IST [8].

In our study the clonal evolution was occurred in 4 patients. In a retrospective study on 840 AA patients [20], the incidence of MDS/AML was 10.9% at 10 years after IST while Li et al [21] in a study on 802 AA patients reported a 2.5% incidence of MDS/AML at 10 years after IST. The discrepancy in incidence of clonal evolution might be due to different patient populations, the diagnostic criteria and treatment protocols.

The main strength of this study is the long-term period of the patients^,^ follow up. During the 15 years, the first author monitored the process of diagnosis, treatment and follow-up of all patients and the criteria for diagnosis, response and relapse did not change in our hospital. The retrospective nature is the main limitation of our study.

Our results indicate that patients with AA which were unable to receive HSCT could be treated by IST with comparable results. Also, our experience indicated that IST should not be discontinued after response to therapy in patient with both NSAA and SAA due to high risk of relapse. Low dose of CsA should be continued indefinitely. Every case should be followed for life because very late relapse is also possible.

## Supporting Information

S1 Table Individual characteristics of 43 patients who were at risk of relapse. Characteristics of patients who responded to treatment.
(DOCX)
