# Supplementary material for: Immunosuppressive Therapy in Patients with Aplastic Anemia: A Single-Center Retrospective Study
Source: PLoS One. 2015 May 13;10(5):e0126925. doi: 10.1371/journal.pone.0126925 (PMC4430492; doi:10.1371/journal.pone.0126925)
Supplement: S1 Table — Characteristics of patients who responded to treatment. (DOCX) [file pone.0126925.s001.docx]

| Individual characteristics of 43 patients who were at risk of relapse. | | | | | | | | | | |
| --- | --- | --- | --- | --- | --- | --- | --- | --- | --- | --- |
| No. | Sex | Age at diagnosis (years) | Etiology | Type of AA | Type of treatment | Discontinuation of CsA | Response at 3 months | Response at 6 months | Relapse | Time of relapse after treatment (months) |
| 1 | M | 35 | Idiopathic | SAA | CsA+D |  | PR | PR |  |  |
| 2 | F | 28 | Idiopathic | NSAA | CsA+D |  | NR | PR |  |  |
| 3 | M | 39 | Idiopathic | SAA | CsA+D |  | PR | PR |  |  |
| 4 | F | 36 | Idiopathic | SAA | CsA+D |  | PR | PR |  |  |
| 5 | M | 20 | Chemicals | SAA | CsA+D | Discontinued | NR | PR | Relapsed | 85 |
| 6 | M | 19 | Idiopathic | SAA | ATG+CsA+D | Discontinued | PR | CR | Relapsed | 37 |
| 7 | M | 23 | Chemicals | NSAA | ATG+CsA+D |  | NR | NR |  |  |
| 8 | F | 14 | Idiopathic | NSAA | CsA+D | Discontinued | NR | PR |  |  |
| 9 | M | 39 | Idiopathic | SAA | CsA+D |  | NR | NR |  |  |
| 10 | M | 54 | Idiopathic | NSAA | CsA+D | Discontinued | CR | CR |  |  |
| 11 | M | 37 | Idiopathic | NSAA | ATG+CsA+D |  | NR | CR |  |  |
| 12 | F | 17 | Idiopathic | NSAA | CsA+D |  | NR | PR |  |  |
| 13 | F | 18 | Parvovirus B19 | NSAA | CsA+D |  | PR | CR |  |  |
| 14 | F | 21 | Idiopathic | SAA | CsA+D |  | NR | NR |  |  |
| 15 | M | 21 | Idiopathic | SAA | CsA+D |  | NR | PR |  |  |
| 16 | M | 20 | Idiopathic | NSAA | CsA+D |  | PR | PR |  |  |
| 17 | F | 34 | Idiopathic | SAA | CsA+D | Discontinued | NR | PR | Relapsed | 66 |
| 18 | F | 24 | Idiopathic | SAA | ATG+CsA+D | Discontinued | NR | CR | Relapsed | 53 |
| 19 | M | 35 | Idiopathic | NSAA | CsA+D |  | NR | CR | Relapsed | 47 |
| 20 | F | 32 | Idiopathic | SAA | CsA+D |  | NR | PR |  |  |
| 21 | M | 52 | Idiopathic | NSAA | CsA+D |  | PR | CR |  |  |
| 22 | M | 20 | Parvovirus B19 | NSAA | CsA+D |  | PR | PR |  |  |
| 23 | M | 40 | Idiopathic | NSAA | CsA+D | Discontinued | NR | PR |  |  |
| 24 | M | 18 | Idiopathic | SAA | CsA+D |  | NR | PR |  |  |
| 25 | F | 28 | Idiopathic | SAA | CsA+D |  | NR | PR |  |  |
| 26 | M | 35 | Idiopathic | SAA | CsA+D |  | NR | PR | Relapsed | 32 |
| 27 | F | 30 | Idiopathic | SAA | CsA+D |  | NR | NR |  |  |
| 28 | M | 20 | Idiopathic | NSAA | ATG+CsA+D |  | NR | PR |  |  |
| 29 | F | 26 | Idiopathic | NSAA | CsA+D |  | PR | CR | Relapsed | 16 |
| 30 | M | 49 | Hepatitis | NSAA | CsA+D | Discontinued | CR | CR |  |  |
| 31 | F | 31 | Idiopathic | NSAA | CsA+D |  | PR | PR |  |  |
| 32 | M | 30 | Idiopathic | NSAA | ATG+CsA+D |  | NR | PR |  |  |
| 33 | M | 25 | Hepatitis | SAA | ATG+CsA+D |  | NR | NR |  |  |
| 34 | M | 23 | Idiopathic | SAA | CsA+D |  | NR | PR |  |  |
| 35 | M | 22 | Idiopathic | NSAA | CsA+D | Discontinued | PR | PR |  |  |
| 36 | F | 45 | Idiopathic | NSAA | CsA+D | Discontinued | PR | PR | Relapsed | 78 |
| 37 | M | 22 | Idiopathic | NSAA | CsA+D | Discontinued | PR | PR | Relapsed | 60 |
| 38 | M | 19 | Idiopathic | NSAA | CsA+D | Discontinued | PR | CR | Relapsed | 118 |
| 39 | F | 34 | Idiopathic | NSAA | CsA+D |  | PR | CR |  |  |
| 40 | F | 57 | Idiopathic | NSAA | CsA+D |  | PR | PR |  |  |
| 41 | F | 15 | Idiopathic | NSAA | CsA+D |  | PR | CR |  |  |
| 42 | M | 38 | Idiopathic | NSAA | CsA+D |  | CR | CR |  |  |
| 43 | M | 18 | Idiopathic | NSAA | CsA+D |  | PR | PR |  |  |

F female, M male, SAA severe aplastic anemia, NSAA non-severe aplastic anemia, ATG antithymocyte globulin, CsA cyclosporine A, D danazol, NR no response, PR partial response, CR complete response
